# The effect of high and low velocity-based training on the throwing performance of collegiate handball players

**DOI:** 10.7717/peerj.14049

**Published:** 2022-09-28

**Authors:** Bassam Abuajwa, Mike Hamlin, Eliza Hafiz, Rizal Razman

**Affiliations:** 1 Centre for Sport & Exercise Sciences, University of Malaya, Kuala Lumpur, Kuala Lumpur, Malaysia; 2 Department of Tourism, Sport and Society, Lincoln University, Christchurch, Canterbury, New Zealand

**Keywords:** Velocity-based training, Strength training, Bench press, Handball throwing, Athletic performance

## Abstract

**Background:**

The intensity of strength training exercise is generally regarded to be the most essential element in developing muscle strength and power. The exercise intensity of strength training is known as one-repetition maximum (1RM). Velocity-based training (VBT) has been proposed as a different approach for determining training intensity. VBT relies on the use of linear position transducers and inertial measurement units, providing real-time feedback to objectively adjust the exercise intensity based on an athlete’s velocity zone.

**Methods:**

This study investigated the effects of two different training interventions based on individualized load velocity profiles (LVP) on maximal bench press strength (*i.e.*, 1RM), maximum throwing velocity (TV), and skeletal muscle mass (SKMM). Twenty-two university handball players were randomly assigned to Group 1 (low-movement speed training) or Group 2 (high-movement speed training). Group 1 exercised with a bar speed of 0.75–0.96 m/s, which corresponds to a resistance of approximately 60% 1RM, whereas Group 2 trained at 1.03–1.20 m/s, corresponding to a resistance of approximately 40% 1RM. Both groups exercised three times a week for five weeks, with strength and throwing tests performed at baseline and post-intervention.

**Results:**

A two-way repeated measures ANOVA was applied, and the results showed the interaction between group and time was not statistically significant for SKMM (*p* = 0.537), 1RM (*p* = 0.883), or TV (*p* = 0.774). However, both groups significantly improved after the five weeks of training: SKMM (3.1% and 3.5%, *p* < 0.01), 1RM (15.5% and 15.0%, *p* < 0.01), and throwing velocity (18.7% and 18.3%, *p* < 0.01) in Group 1 and 2 respectively. Training at both prescribed velocities in this study elicited similar changes in strength, muscle mass, and throwing velocity.

## Introduction

Previous research has shown a close relationship between upper body strength and throwing performance (Hermassi et al., 2010; Vanden Tillaar, 2004). Strength training is a fundamental stimulus for enhancing muscular strength, size, and power; exercise intensity is an essential stimulus associated with a change in strength level and commonly identified with relative load (percentage of one-repetition maximum, % 1RM) (Jovanović & Flanagan, 2014). However, to improve power in strength training programs, there is always a conflict between using heavier or lighter resistance load. The use of heavier resistance (80–100% 1RM) based on the size principle, recruits high-threshold fast-twitch motor units (McBride et al., 2002). Alternatively, with lighter resistance (30–40% 1RM) larger velocities and accelerations may be attained, resulting in a greater transfer of training impacts to those athletic activities (Crewther, Cronin & Keogh, 2005).

There has recently been considerable concern regarding percentage-based training (PBT); some researchers discourage the direct determination of 1RM since it requires more time to process (Rauch et al., 2018), and is impractical for large groups (Marcos-Pardo et al., 2019). Moreover, the outcome can be affected by different variables, including the athlete’s experience level, lifting form and technique, and the motivation for the performance (McMaster et al., 2014). Furthermore, because maximum strength fluctuates daily due to fatigue levels, it may not accurately reflect an athlete’s current capabilities (Blazevich et al., 2007). The disadvantages mentioned above allow for new methods to objectively measure and modify training loads during resistance training.

However, one study by (Marques et al., 2007), used alternative approach which does not require determination of 1RM, used three fixed loads (26–36–46 kg) to determine power production and bar velocity in bench press, executed by group of elite handball players. There are few studies that show a very high variation between individuals (Pareja Blanco et al., 2020), which affects the level of effort undertaken by individuals, consequently decreasing the lifting velocity repetition during the set (Sanchez-Medina & González-Badillo, 2011). The growing availability of technology in sports, such as linear position transducers and inertial measurement units (Guerriero, Varalda & Piacentini, 2018), enables load modification daily to match desired intensity (*i.e.*, % 1RM).

Velocity-based training (VBT) is an indirect method that relies on the load-velocity relationship to predict 1RM in resistance training, which has the advantage of monitoring exercise intensity (Gonzalez-Badillo & Sanchez-Madine, 2010). Moreover, the force-velocity relationship reflects the neuromuscular system’s ability to perform under different training load conditions and thus significantly affects movement (Cronin, McNair & Marshall, 2003). The intention to move explosively is considered fundamental for a high-velocity-specific response to resistance training, and it has been suggested that the intention to move quickly defines the velocity-specific response (Behm & Sale, 1993).

Several studies suggest using VBT to monitor the training load objectively throughout the training session (Gonzalez-Badillo & Sanchez-Madine, 2010), while taking into consideration the athlete’s daily fluctuations in performance or fatigue levels (Balsalobre-Fernández & Torres-Ronda, 2021). A study by Rauch et al. (2018) compares two velocity approaches, the progressive velocity-based training (PVBT) used a movement speed range of 0.55–1 m/s, while the other group trained with optimal training load with 0.85 m/s for seven weeks. The findings showed that both training groups demonstrated muscle and power improvement in female volleyball players.

Interestingly, a few studies demonstrated strength gains but no improvement in throwing velocity in handball. One study (Medica, 2020) compared upper body bench press on throwing velocity over four weeks, with 30 subjects divided into two groups. The strength group trained at 70–90% 1RM, and the ballistic group trained at 40% 1RM. Neither group improved throwing velocity. Similarly, a study by Cuevas-Aburto et al. (2020) compared the effect of two distinct bench press exercises on handball throwing velocity: 1RM bench press, bench throw, bench press throw peak velocity with load of 30 kg. Both groups trained two days a week for six weeks, including bench press throw peak velocity with a load of 30 kg, and bench press with a load. Both groups trained with load of 75% 1RM with different set configurations. The results of this study showed improvement in 1RM strength and bench press throw but did not show any improvement in throwing velocity.

However, mean concentric velocity (MCV) and load (%1RM) are related; the relationship is exercise-dependent, as the load that represents an absolute velocity can vary considerably across training (Balsalobre-Fernández & Torres-Ronda, 2021). Importantly, because of the wide variation among individuals in the velocity with a particular %1RM, the research indicates that individualized load velocity profiles need to be established rather than group velocity zones (Balsalobre-Fernández, García-Ramos & Jiménez-Reyes, 2018; García-Ramos et al., 2018a). However, it is questionable whether training to move explosively during either heavy or light loads will adapt to actual sports performance for a precise and objective prescription of resistance training. Moreover, there is a shortage of data regarding the applicability of individualized load velocity profile (LVP) to sports performance skills such as throwing velocity.

The unique aspect of the current study is the application of VBT to individualize and adjust training load for each participant based on the individualized LVP. Therefore, the purpose of this study is to investigate the changes in strength and throw performance in college handball players after five weeks of strength training using movement velocity as the independent variable of %1RM, on maximal bench press strength (1RM), maximum throwing velocity (TV), and skeletal muscle mass (SKMM) in university handball players. The application of VBT in this study allows for the use of individual LVPs to exert force at speeds specific to the load during a sessional target velocity zone, at the same time, using real-time velocity feedback to dictate the load adjustment set-by-set throughout the training session. The two training velocities are low movement velocity and high movement velocity.

## Materials & Methods

### Participants

A total of 22 male active collegiate handball players were recruited for this study (age, 19.86 ± 0.83 years; height, 171.36 ± 4.58 cm; weight, 67.68 ± 11.21 kg; and muscle mass, 31.01 ± 4.51 117 kg). The participants were randomly assigned into two groups: Group 1 (low-movement velocity), which exercised with a bar speed of 0.75–0.96 m/s, that corresponds to a resistance of approximately 60% 1RM; and Group 2 (high-movement velocity), which trained at 1.03–1.20 m/s, corresponding to a resistance of approximately 40% 1RM. The inclusion criteria for this study were: (a) familiarity with the exercises in the experiment, with at least six months of experience in resistance training for upper limb strength; (b) being between the ages of 18 and 30 years old, and (c) participants were also required to be highly active (3,000 MET minutes a week or higher based on the International Physical Activity Questionnaire. Exclusion criteria were: (a) previous injury such as musculoskeletal injuries affecting training in the current study, or disease that might interfere with the study and (b) on medication or steroids that could improve performance. All participants were injury-free at the time of testing and were also required to refrain from any other form of physical activity or exercise during the study; participants continued the handball training only. Prior to participation in this study, the subjects were briefed about the protocol, and participants read and signed the informed consent form that was approved by the University of Malaya Research Ethics Committee (Reference Number: UM.TNC2/UMREC-403).

### Procedures

This was an experimental study whereby participants in both groups completed five weeks of bench press exercise training. A few studies show that short-time resistance training provides different effects on explosive strength and agility indicators over five weeks (Tomljanović et al., 2011), or provides a similar strength improvement in VBT against traditional resistance exercise over a duration of four weeks (Pelka & Claytor, 2019), or the effectiveness of traditional resistance exercise over five weeks (Montalvo-Pérez et al., 2021).

The pre- and post-tests included body composition measurement, %1RM assessment, LVP assessment, and a handball throwing velocity test. Participants did not participate in any physical exercise for one day prior to testing. All tests were performed on a single day and under the same experimental conditions. There was also a familiarization session prior to the testing day before commencing the training. The intervention training frequency was three days a week over five weeks, separated by at least 48 h of recovery, in both training groups.

### Body composition assessment

All participants had their height measured on a Seca scale (model: seca763, Germany), while body weight and muscle mass were measured using the InBody machine (InBody370 model: JMW140; Biospace, Seoul, South Korea), using standard testing procedures prior to breakfast and after the participants had emptied their bladders. Bioelectrical impedance analysis (BIA) offers a low-cost, easy, and safe alternative to DEXA to provide body composition data with appropriate accuracy and reliability (Larsen et al., 2021). In addition, it has been used to evaluate the efficacy of an upper-body strength training program on bench press 1RM, peak velocity in bench throws, and handball throwing velocity (Cuevas-Aburto et al., 2020).

### Bench press

Bench press exercise was used in this experiment because it has a highly specific relationship with overhand throwing (Hermassi et al., 2010), and may allow researchers readily to evaluate maximal upper body strength and throwing performance (Ettema, Gløsen & vandenTillaar, 2008). The bench press procedure started with the participant lying supine on the bench of the Smith Machine (Body-Solid 7 Linear Bearing Smith Machine; Body-Solid, Kuala Lumpur, Malaysia). The advantage of using the Smith Machine is that the bar is attached at both ends, enabling balanced vertical movement and more control (Hermassi et al., 2019). In addition, it provides steady motion to less-experienced subjects (Schick, 2009), and Mann (2016) suggests executing stable and controlled movement during VBT training, since participants try to achieve the highest velocity which may affect performance technique.

The participant lowered the bar until it lightly touched his chest, holding it there for 2–3 s to eliminate any excess energy from the eccentric phase and to minimize the rebound effect to allow for more consistent measurement. The hips and feet remained in contact with the bench and the floor, respectively. The participants were instructed to execute the rep as quickly as possible while extending their elbows and flexing their shoulders. During training, the velocity of each lift was monitored and recorded by an accelerometer attached to the bar, and the weight was adjusted during training based on the barbell velocity after each set to meet the velocity target.

### Bench press-one repetition maximum (1RM) assessment

Participants were instructed to control the eccentric phase before pushing the bar during the concentric phase. The participants completed the first set with an empty bar and a range of 8–10 repetitions, followed by five repetitions that were 50% of the estimated 1RM, then one set of 3–5 repetitions with an estimated 60–70% 1RM, and one set where the first repetition attempt was 90% of an estimated 1RM.The 1RM was recorded for the maximum successfully lifted weight, with the 1RM attempt within 3–5 sets with 5-minute intervals between 1RM attempts, as recommended by the National Strength and Conditioning Association (Dorrell, Smith & Gee, 2020). Verbal encouragement and velocity feedback were given to motivate the subjects to exert their maximum effort: both groups were instructed to exert the maximum effort for each repetition regardless of the weight and to maintain a steady, correct technique.

### Load velocity profile (LVP) assessment

Velocity-based training involves modifying the training loads to a specific velocity target, which is created via an individualized LVP regression equation (Banyard et al., 2019). The 1RM evaluation allows for a precise LVP assessment (Jidovtseff et al., 2011). LVP was tested two days after the 1RM session. The subjects performed five incremental loading tests between 20–90% of 1RM, utilizing a linear regression equation to construct the LVP to apply a line of best fit and to convert relative load percentage (kg) against movement velocity (m/s). The five incremental loads included three repetitions for 20%, 40%, and 60% 1RM, as well as one repetition for 80% and 90% 1RM, with two minutes’ rest between attempts. The LVP was determined using the fastest repetitions in each load (Balsalobre-Fernández & Torres-Ronda, 2021; Weakley et al., 2021).

Due to the study’s focus on the effect of training velocity on performance, after the LVP assessment, the two groups were randomly assigned to the two different velocity zones at each load (low-movement velocity of 0.75–0.96 m/s, and high-movement velocity of 1.03–1.20 m/s). The randomization process used the random sampling as it considers the easiest approach, which involve placing the participants’ names on cards in a container and then picked a card until the groups are achieved (Gratton & Jones, 2010). This enabled us to adjust the velocity of movement throughout training. Randomization was used to eliminate bias and ensure that groups were evenly matched, as well as to differentiate the training velocity based on the recommendation by Banyard et al. (2020).

### Velocity monitoring device

The Beast sensor (Beast Technologies S.r.l., Brescia, Italy) wearable wireless accelerometer is comprised of a three-axis accelerometer, three gyroscopes, and a three-axis magnetometer with an acquisition frequency of 50 Hz; the device has shown good reliability and validity during bench press, full squat, and hip thrust exercises (Balsalobre-Fernández et al., 2017).The Beast sensor was attached to the bar (built-in magnet) throughout the session to ensure the training was appropriate with all the velocity zones. The unit was linked to an iOS-based smartphone via Bluetooth 4.0 Low Energy, using Beast strength application version 2.4.4. The MCV throughout each exercise was recorded, and the load was adjusted according to the desired speed.

### Training intervention

The Beast sensor recorded each repetition and provided visual and auditory feedback in real-time. A screen was placed in front of the participant to provide immediate visual feedback, enabling them to adjust their concentric lifting velocity to their individual velocity zone. The LVP for participants regulated the load based on the velocity of the movement for each set, which means the participants, regardless of which training group they were in, moved the bar as quickly as possible for each repetition. Participants performed warm-up sets and training sets as described below.

### Warm-up set

The participants performed the same warm-up routine before training for each session. This routine included a monitored 15-minute standard warm-up session consisting of 20 sit-ups, five minutes of dynamic joint mobilization work in the upper limbs, and 10 push-ups. Participants completed three warm-up sets while their movement velocity was monitored by the Beast sensor, consisting of 20% 1RM (3 repetitions) and 40% 1RM (3 repetitions) for the high movement speed group, and 60% 1RM (3 repetitions) for the low-speed group, followed by one repetition performed in the required velocity target of each group, which was determined using a participant’s individualized LVP. The rest between the warm-up set and training set was 2 min.

### Training set

The participants completed six repetitions in four sets, with the external load adjusted by mean concentric velocity (MCV). If the last set average at the warm-up set was ± 0.06 m/s higher or lower than the target velocity from LVP, then the first training set would be modified by ± 5% 1RM (Banyard et al., 2019; Weakley et al., 2021). Similarly, if the average of the first set was ± 0.06 m/s higher or lower than the target velocity, the load was adjusted by ± 5% 1RM for the second set. In this manner, the participants completed the training according to their velocity target during the session, as shown in Table 1.

**Table 1:** Load adjustment based on the individualized velocity zone for each player in two groups.

	Group 1 (0.75–0.96 m s^−1^, 60% 1RM)			Group 2 (1.03–1.20 m s^−1^, 40% 1RM)		
Player	Load velocity profile (LVP) (m s^−1^)	Velocity zone (± 0.06 m s^−1^)	Equivalent Load ± 5% 1RM (kg)	Load velocity profile (LVP) (m s^−1^)	Velocity zone (±0.06 m s^−1^)	Equivalent load ± 5%1RM (kg)
1	0.87	(0.81–0.93)	28.5 (23.5–33.5)	1.06	(1.0–1.12)	20 (15–25)
2	0.82	(0.76–0.88)	40 (35–45)	1.16	(1.10–1.22)	16 (11–21)
3	0.88	(0.82–0.94)	30 (25–35)	1.2	(1.14–1.26)	20 (15–25)
4	0.9	(0.84–0.96)	30 (25–35)	1.05	(0.99–1.11)	38 (33–43)
5	0.75	(0.69–0.81)	30 (25–35)	1.09	(1.03–1.15)	18 (13–23)
6	0.77	(0.71–0.83)	24 (19–29)	1.1	(1.04–1.16)	18 (13–23)
7	0.9	(0.84–0.96)	30 (25–35)	1.03	(0.97–1.09)	24 (19–29)
8	0.81	(0.75-0.87	39 (34–44)	1.13	(1.07–1.19)	22 (17–27)
9	0.8	(0.74–0.86)	42 (37–47)	1.03	(0.97–1.09)	29 (24–34)
10	0.93	(0.87–0.99)	30 (25–35)	1.11	(1.05–1.17)	24 (19–29)
11	0.96	(0.90–1.02)	54 (49–59)	1.18	(1.12–1.24)	20 (15–25)

### Ball-throwing velocity

The participants performed the test by throwing a standard handball (Size 3, circumference 58–60 cm, weight 435 g) towards a goal. After a standardized warm-up of 10 min, players threw at maximum velocity using their dominant arm while in a standing throw stance from a 7-meter line with the front foot on the ground at all times. The instruction was to throw as fast as possible while targeting the middle of a handball goal with three minutes’ rest between throws. The three fastest throws out of five were used to calculate the mean velocity. Ball movement was recorded at 240 frames per second at 1,080 pixels in high definition using an iPhone 11. The camera was placed perpendicular to the line between the participant and the center of the goal. Previous research has shown that between 120 and 240 frames per second was suitable for performing velocity measurements in sports (Quintana & Padullés, 2016). The ball velocity was derived from the distance traveled during the first five frames after the ball’s release. The ball-throwing velocity videos were analyzed using Kinovea (0.8.15. Dnu general public license Version 2). Kinovea is a 2D-motion analysis software that allows for the determination of kinematic parameters and is a validated tool for assessing time-related variables (Puig-Diví et al., 2017).

### Statistical analysis

A general linear model with Bonferroni post hoc correction was applied to determine the difference between and within groups. A two-way repeated-measures ANOVA was applied to determine significant differences between the groups, and the criterion level was set at P 0.05. All the assumptions, such as normality and outliers, were analyzed by using the Shapiro–Wilk test. The power calculation was carried out with G*Power software (3.1.9.7), using a post hoc test. Effect size was calculated using mean and standard deviations. All statistical analyses were carried out using SPSS software (Version 25; SPSS, Inc., Chicago, IL, USA).

Previous research with similar sample sizes has shown that VBT positively impacts the development of muscle strength, power, and throwing velocity with the absence of a control group, but especially in those involving competitive athletes (Rauch et al., 2018) in volleyball and futsal (Marques et al., 2019), or competitive female cyclists (Montalvo-Pérez et al., 2021).

## Results

The standard deviations and percentages of change for both groups are presented in Table 2. In the low-velocity training group (G1), SKMM increased 3.12%, from pre-intervention (30.9 ± 4.37 kg) to post-intervention (31.86 ± 4.07 kg). Similarly, 1RM improved 15.48%, from 57.27 ± 14.33 kg to 67.71 ± 14.42, as did TV (18.70%, from 14.77 ± 2.11 pre-training to 18.41 ± 3.45 m/s post-training).

**Table 2:** Means (± SD), along with the percentage change in muscle mass, 1RM, and ball throwing speed for both groups.

Group		Pre-Test (Mean ± SD)	Post-Test (Mean ± SD)	Percent Change
Low velocity movement	Muscle Mass	30.9 ± 4.37 kg	31.86 ± 4.07 kg	3.12%
	%1RM	57.27 ± 14.33 kg	67.71 ± 14.42 kg	15.48%
	Ball Throwing velocity	14.77 ± 2.11 m.s^−1^	18.41 ± 3.45 m.s^−1^	18.70%
High velocity movement	Muscle Mass	31.12 ± 4.87 kg	32.37 ± 5.76 kg	3.54%
	%1RM	56.59 ± 15.58 kg	67.5 ± 21.30 kg	15.02%
	Ball Throwing velocity	14.21 ± 2.78 m.s^−1^	17.55 ± 3.18 m.s^−1^	18.32%

The mean values of the high-velocity group (G2) are: SKMM increased 3.54%, from pre-intervention (31.12 ± 4.87) to post-intervention (32.37 ± 5.76), 1RM improved by 15.02%, from 56.59 ± 15.58 kg to 67.5 ± 21.30 kg, while TV increased 18.32%, from 14.21 ± 2.78 m/s pre-training to 17.55  ± 3.18 m/s post-training.

There was a significant difference between pre-test and post-test for all the variables for each group separately. Pre-test and pos *t*-test data in both groups have similar changes: for SKMM G1, *p* = 0.006, and G2, *p* = 0.001; 1RM for G1, *p* ≤ 0.001, and G2, *p* = <0. 001; and TV G1, *p* ≤ 0.001, and G2, *p* ≤ 0.001.

The results showed significant changes between pre- and post-tests for SKMM (F (1, 20) = 24.298. *p* < 0.001, ⋅2 = 0.549), 1RM (F (1, 20) = 48.926, *p* < 0.001, ⋅ 2 = 0.710), and TV (F (1, 20) = 43.854, *p* < 0.001, ⋅2 = 0.687).

The comparison between groups at pre-test and pos *t*-test, however, indicated there were no significant differences between the two groups.

G1 (low-velocity group): SKMM, pre-test *p*-value = 0.909, and post-test *p*-value = 0.813, an estimate of effect size = 0.22 at observed power of 16.7%. The 1RM *p*-value for pre-test = 0.916 and post-test *p*-value = 0.977, an estimate of effect size = 0.72 at observed power of 71.8%. For TV, the pre-test *p*-value = 0.606, and post-test *p*-value = 0.550, an estimate of effect size = 1.27 at an observed power of 98.8%.

In G2 (high-velocity group), SKMM showed an estimate of effect size = 0.23 at an observed power of 17.5% 1RM, with an estimate of effect size = 0.58 at an observed power of 55.8%. For TV, an estimate of effect size = 1.12 at an observed power of 96.4%.

In summary, the comparison between groups at pre-test and pos *t*-test indicated there was no significant difference between the two groups. These results indicate that the interaction between group and time was not statistically significant: SKMM (F (1, 20) = 0.395, *p* = 0.537, *η*^2^ = 0.019), 1RM (F (1, 20) = 0.022, *p* = 0.883, *η*^2^ = 0.001), TV (F (1, 20) = 0.085, *p* = 0.774, *η*^2^ = 0.004). See Table 3.

**Table 3:** Result of Bonferroni test for mean comparison for both groups, after five weeks of training in the bench press exercise.

**Variable**	**Group**	**Pre-Test** (mean ± SD)	**Between groupat pre-test***p* Value +	**Post-test** (mean ± SD)	**Between group at post-test***P* value+	**Within group pre-post change***P* Value ++	**Effect size** **(Pre vs. post)**
Muscle mass	Low velocity	30.9 ± 4.37	0.909	31.86 ± 4.07	0.813	0.006^*^	0.22 (small)
	High velocity	31.12 ± 4.87		32.37 ± 5.76		0.001^*^	0.23 (small)
%1RM	Low velocity	57.27 ± 14.33	0.916	67.71 ± 14.42	0.977	<0.001^*^	0.72 (medium)
	High velocity	56.59 ± 15.58		67.5 ± 21.30		<0.001^*^	0.58 (medium)
Throwing velocity (m/s)	Low velocity	14.77 ± 2.11	0.606	18.41 ± 3.45 m/s	0.550	<0.001^*^	1.27 (large)
	High velocity	14.21 ± 2.78		17.55 ± 3.18 m/s		<0.001^*^	1.12 (large)

**Notes.**

* Significant at 0.05 level.

+ between group comparison.

++ Within group comparison.

## Discussion

This study investigated handball players’ strength and throwing velocity changes after five weeks of different strength training velocity-based loads. There were no significant differences between the two groups, with both groups increasing muscle mass, 1RM, and throwing velocity from pre- to post-intervention. Furthermore, to the author’s knowledge, this is the first study to investigate the effects of velocity-based training on throwing velocity using individualized load velocity profiles.

Our findings suggest that both groups significantly improved between pre-test and post-test for all variables after the five weeks of training.The speed of movement may be improved by a heavy resistance training load in which the actual movement velocity is low, although the intention is to lift weight as rapidly as possible, according to previous studies (*e.g.*, Behm & Sale, 1993; Cronin, McNair & Marshall, 2001).

On the other hand, the ability of neuromuscular systems to produce maximal power production is based on training with light loads (Rahmani et al., 2001), which appears to be crucial in sports movements such as sprinting, jumping, or throwing, where force output is required over a short period of time (Newton & McEvoy, 1994). A study of male handball players (Gorostiaga et al., 2005) who trained with a velocity at 30% 1RM showed improvement in the athletes’ throwing velocities for the standing throw, indicating that maximal strength performance is not always related to ball-throwing velocity capability. This possibly explains that, regardless of handball skill level, high-velocity training with a light load during bench press action may result in a higher throwing velocity from a standing position (Gorostiaga et al., 2005).

The main finding of this study was the high-movement velocity group; the results showed that the movement speed the high-velocity group trained at (1.03–1.20 m/s) corresponded to a 40% 1RM, showing great strength gain in the bench press and throwing performance (see Table 3). The increase in muscle mass after training with high-movement velocity with a light load could be explained by an increase in the muscle cross-sectional area, as a previous study showed a high correlation between cross-sectional area and muscle strength when using a lighter weight (Moss et al., 1997). In addition, several studies have found that high-velocity training enhances performance and increases muscle power more than low velocity (Kawamori & Haff, 2004). As this current study did not use an electromyography device during testing, it is possible to speculate that the high-velocity group stimulated a fast-twitch ability. Others have proposed that rapid movement with low load may be used to recruit and fire fast high-threshold motor units (DeRenne et al., 1994).

Moreover, a previous study showed that the primary determinant of velocity response depends on the intention to move the load rapidly instead of the actual movement performed during the exercise (Behm & Sale, 1993). However, the findings of this study do not agree with that conclusion and showed different training responses between both groups. The result demonstrated the comparison between groups at pre-test and post-test, as both groups in our current study were instructed to perform the movement explosively while monitoring the individualized LVP. The greatest differences were observed in the 1RM test in the effect size, as Group 1 had a relatively larger effect than Group 2 (0.72, 0.58 respectively), and the throwing velocity showed both groups had considerable differences between pre-test and post-test (1.27, 1.12 respectively).

Our results agree with the velocity specificity principle, which states that training-induced adaptations in strength and power are maximized at or near the velocity used during training (Kawamori & Newton, 2006). For example, a study by McBride et al. (2002) demonstrated that over eight weeks, the actual movement velocity could influence the velocity-specific response to resistance training, when compared to the effects of training with a heavy (80% 1RM) load or a light (30% 1RM) load jump squat, both intending to move explosively, and found that the training responses were different between the groups. They found that the light load training group increased peak velocity, peak power, and jump heights during jump squat test, while the heavy load group showed a drastic decline in the sprint test.

The periodized resistance training program can alter the 1RM while the velocity remains stable during training, allowing for the objective monitoring of each participant’s training load to ensure they train at the desired velocity zone (Weakley et al., 2021). Therefore, it is essential to distinguish between the VBT regimen employed in this study and the percentage-based training regimens (PBT) described in most prior studies. For example, a study by Marques & González-Badillo (2006) examined the effects of concentric bench presses at a load of 70–85% 1RM for 12 weeks in 16 high-level handball players and found a 28% increase in 1RM; however, this study did not apply explosive movement velocity during training. Therefore, the limitation of prescribing a load based on 1RM may not reflect the participants’ true strength and could potentially impact the training stimulus and adaptations (Moore & Dorrell, 2020).

Participants in the current study performed maximal intended velocity while referencing the last repetition at the end of the warm-up set to ensure an exact match between the velocity and the %1RM to be utilized during the session based on their LVP. The low-movement velocity group exercised with a bar speed of 0.75–0.96 m/s, corresponding to a resistance of approximately 60% 1RM, and demonstrated an improvement in 1RM of 15.48%, which corresponds with muscle mass improvement (30.9 ±  4.37 kg to 31.86 ± 4.07 kg).

In comparison, the results observed in the slow-velocity group in the study by Hermassi et al. (2010), over a 10-week period that included the bench press, trained with a load of 80–95% 1RM and showed an improvement of 16% 1RM. However, the shorter duration of five-weeks of the present study indicates that the results were obtained with a lower degree of effort while applying the individualized LVP during resistance training (Banyard et al., 2020), yet the heavier loads and the intent to move an external load as fast as possible are critical factors for enhancing strength.

Moreover, a study of volleyball players by Rauch et al. (2018) compared two common approaches in VBT; the progressive overload (PVBT) group, and optimum training loads group (OPL). The groups trained three days a week for seven weeks. The PVBT group used bench press exercises and trained gradually with high force and lower velocity during a strength block (0.55–0.70 m/s), then trained for three weeks of power block (0.85–1.0 m/s). Participants showed an 8.5% improvement in bench press %1RM. The OPL group trained with load to maximize the power output (0.8–1.0 m/s) and adjusted the training load based on the increase or decrease of movement velocity for the required training zone, showed improvement of 10.2% in bench press %1RM. Additionally, both training methods have been shown to enhance muscle strength, power development, and muscle mass in female volleyball players. In our present study, the group that trained with 0.75–0.96 m/s showed bench press 1RM improved 15.48% for the low-velocity. However, the low velocity group in our current study was similar to the PVBT approach by Rauch et al. (2018) which trained at a speed of 0.55–1.0 m/s. Moreover, the high-velocity group in our study, however, trained with a speed of 1.03–1.20 m/s showed bench press 1RM improved by 15.02%.

In regards to throwing velocity, a study by Medica (2020) compared upper body bench press on throwing velocity over four weeks, with 30 subjects divided into two groups, the strength group (STG) and the ballistic group (BTG). The STG trained with 70–90% 1RM, and the BTG trained at 40% 1RM. Neither group showed improved throwing velocity. The STG enhanced the 1RM effect size (ES:0.24), and no improvement in handball throwing velocity (ES = 0.10), while the BTG did not improve the %1RM (ES = 0.27) or handball throwing velocity (ES = 0.02). However, in our current study, the throwing velocity improved in both groups: the low-velocity group trained with 0.75–0.96 m/s, showed 1RM (ES: 0.72), while the throwing velocity (ES = 1.27). The high-velocity group showed1 RM (ES = 0.58), while throwing velocity (ES = 1.12).

Other studies showed strength gains but no improvement in throwing velocity in handball. A study (Cuevas-Aburto et al., 2020) compared the effect of two distinct bench press exercises trained for two days a week for six weeks on handball throwing velocity, 1RM bench press and bench throw, as one group performed five repetitions during six sets with a longer interest -rest period of 3 min, while the other group performed one set of 30 repetition with 31 s of inter-repetition rest, with load 75% 1RM.Furthermore, Participants were instructed to perform with maximal velocity during training. The results showed improvement in 1RM strength and bench press throw, they did not show any improvement in throwing velocity. Our current findings demonstrate that the individualized LVP can enhance velocity specificity principles.

The strength finding in our study shows that the load velocity profile was created for individuals. The existing relationship between velocity (m/s) and load (kg) highlighted the application of individualized LVP in strength fields (Sánchez-Medina et al., 2014; Torrejón et al., 2019). Furthermore, the LVP is time-efficient and has demonstrated the accuracy of estimating 1RM. In addition, the LVP does not appear to change due to the strength level between individuals of the same age (Sánchez-Medina et al., 2017). Previous studies showed that the barbell velocity that corresponds with the load 1RM in resistance training does not apply to different muscle groups or to different types of individuals (Fernandez Ortega et al., 2022), as the assessment of LVP depends more on the experience level in strength training, which might be different between individuals on the same team (Fahs, Blumkaitis & Rossow, 2019).

Nevertheless, even though VBT could be useful in terms of strength, previous studies on this method have focused on maximum concentric velocity rather than optimal load prescription (González-Badillo et al., 2015; González-Badillo et al., 2014; Negra et al., 2016). A critical practical application of this study’s method is monitoring the actual load (%1RM) in real-time by measuring the repetition velocity during training. It has also been proposed that real-time velocity feedback allows insight into the players’ current physiological status and readiness to train (González-Badillo et al., 2015). Furthermore, the current study also used mean concentric velocity (MCV) to assess the training load. MCV is thought to better represent the relationship between the relative load and individual effort during non-ballistic movements (back squat, bench press, and bench pull). Still, there is no difference between the mean velocity and mean propulsive velocity MPV (Banyard et al., 2017; García-Ramos et al., 2018a; García-Ramos et al., 2018b; Jidovtseff et al., 2011; Mann, Ivey & Sayers, 2015).

As a result, it is plausible that the instruction for all participants to perform the training with maximum intent during the bench press increased muscle activation and firing frequency, contributing to an increase in the rate of force development, which resulted in an increase of the 1RM (Banyard et al., 2020; Schuenke et al., 2012). Therefore, the addition of LVP enhanced the relative tension in the muscle sufficiently to stimulate absolute strength improvements due to the high force applied during training; this is speculation because the training force was not measured. Another factor that led to the significant improvement in the VBT during resistance training was the instant feedback, which enhanced motivation and allowed for the maintenance of the desired movement velocity level (Randell et al., 2011).

The similar improvement by the two groups of this study highlights the importance of moving quickly in concentric phase training. It has also been suggested that moving explosively may offer significant benefits in training adaptations (Aagaard et al., 2000; Westing, Cresswell & Thorstensson, 1991). Additionally, a few studies suggest inducing a neuromuscular adaptation to resistance training, as both the intention to move explosively and the actual movement are essential (Kawamori & Newton, 2006). Other research has found that resistance training should be accompanied by the practice of actual sport movement to gain the full benefits of training-induced neuromuscular adaptations. For example, Blazevich & Jenkins (2002) reported similar and significant improvement when subjects trained explosively through the practice of sprinting, in two different training groups of elite junior sprinters who had previous resistance training experience (one group at 30–50% 1RM and the other at 70–90% 1RM).

Finally, a limitation of this study is the absence of a control group; however, several applied studies did not use control groups. Previous research has shown that VBT positively impacts the development of muscle strength and power and throwing velocity, especially with competitive athletes in volleyball (Rauch et al., 2018) and futsal (Marques et al., 2019), or competitive female cyclists (Montalvo-Pérez et al., 2021). Moreover, according to Marques & González-Badillo (2006), higher-level players can be affected when placed in a control group and extracting their best performance levels is challenging. Moreover, another limitation is the short duration of our current study (5 weeks/15 sessions), which nonetheless supported the implementation of individualized LVP to induce beneficial adaptations on upper body strength and throwing velocity.

## Conclusions

Velocity-based training increased %1RM, ball-throwing velocity, and muscle mass over five weeks of bench press exercise. However, there was no significant difference between high-velocity or low-velocity training. This indicates that, regardless of velocity/load, considerable improvements may be accomplished when applying VBT.

The bar velocity and training load were constantly monitored and adjusted throughout the intervention based on the LVP. The availability of monitoring devices as motivational tools for feedback makes it easy to apply the VBT to traditional movement. Reporting the average mean velocity of the set makes it easy to create the individualized LVP and compare it with the prescribed velocity zone, offering a comprehensive and accurate knowledge of the athletes’ current conditions. Thus, movement velocity should be acknowledged as a critical factor in modern strength training and should be used to enhance handball throwing velocity and sports performance in general.

##  Supplemental Information

10.7717/peerj.14049/supp-1 Supplemental Information 1 Raw data

## Additional Information and Declarations

### Data Availability

The following information was supplied regarding data availability: The raw data is available in the Supplementary Files.
